# An Overview of Software Sensor Applications in Biosystem Monitoring and Control

**DOI:** 10.3390/s24206738

**Published:** 2024-10-20

**Authors:** Nasem Badreldin, Xiaodong Cheng, Ali Youssef

**Affiliations:** 1 Department of Soil Science, University of Manitoba, 13 Freedman Crescent, Winnipeg, MB R3T 2N2, Canada; nasem.badreldin@umanitoba.ca; 2 Mathematical and Statistical Methods Group (Biometris), Department of Plant Science, Wageningen University & Research, 6700 AA Wageningen, The Netherlands; xiaodong.cheng@wur.nl; 3 Adaptation Physiology Group, Wageningen University & Research, P.O. Box 338, 6700 AH Wageningen, The Netherlands

**Keywords:** software sensors, biosystems, monitoring, control, machine-learning, digital agriculture

## Abstract

This review highlights the critical role of software sensors in advancing biosystem monitoring and control by addressing the unique challenges biological systems pose. Biosystems—from cellular interactions to ecological dynamics—are characterized by intrinsic nonlinearity, temporal variability, and uncertainty, posing significant challenges for traditional monitoring approaches. A critical challenge highlighted is that what is typically measurable may not align with what needs to be monitored. Software sensors offer a transformative approach by integrating hardware sensor data with advanced computational models, enabling the indirect estimation of hard-to-measure variables, such as stress indicators, health metrics in animals and humans, and key soil properties. This article outlines advancements in sensor technologies and their integration into model-based monitoring and control systems, leveraging the capabilities of Internet of Things (IoT) devices, wearables, remote sensing, and smart sensors. It provides an overview of common methodologies for designing software sensors, focusing on the modelling process. The discussion contrasts hypothetico-deductive (mechanistic) models with inductive (data-driven) models, illustrating the trade-offs between model accuracy and interpretability. Specific case studies are presented, showcasing software sensor applications such as the use of a Kalman filter in greenhouse control, the remote detection of soil organic matter, and sound recognition algorithms for the early detection of respiratory infections in animals. Key challenges in designing software sensors, including the complexity of biological systems, inherent temporal and individual variabilities, and the trade-offs between model simplicity and predictive performance, are also discussed. This review emphasizes the potential of software sensors to enhance decision-making and promote sustainability in agriculture, healthcare, and environmental monitoring.

## 1. Introduction

In general, “Biosystems” refers to complex living systems that involve biological components (e.g., cells, tissues, organs, whole organisms, and ecosystems) interacting with each other and their surrounding (micro-)environment [1]. In other words, as per Ruth and Hannon [2], biosystems are systems in which the crucial part of their underlying processes is a living organism, serving as the protagonist that shapes and steers the processes defining these systems. Such systems are complex assemblages of interacting physical, chemical, and biological processes. A significant number of these processes have inherent nonlinearity, and there is substantial uncertainty about their interconnections and nature [3,4]. Additionally, these processes can span the spectrum of scale and complexity, from the microscopic intercellular biological processes like photosynthesis and cellular respiration within a single cell to the macroscopic intricate relationships between living organisms and their environment, such as predator–prey interactions and soil microbiome.

The comprehensive study, understanding, and control of biosystems necessitate watchful monitoring and close observation to unravel their complexity and harness their potential. Therefore, biosystem monitoring plays a decisive role in ensuring the optimal operation of contemporary industrial operations, encompassing agricultural and biological processes. The growing complexity of biological systems in agriculture and environmental conservation has made real-time monitoring more essential than ever, especially as global efforts intensify to improve food security and natural resource sustainability.

Biosystem monitoring involves systematically collecting and analyzing data related to the living organisms involved and their underlying processes. This includes monitoring the living organism’s physiological and behavioral responses. The ultimate goal of biosystem monitoring is to gain insights into the underlying mechanisms, identify potential issues, and optimize conditions for enhanced efficiency and productivity.

In recent decades, there has been a remarkable surge in the application of computational methods to monitor biosystems. This progress has been powered by revolutionary advancements in sensor and sensing technologies, providing us unprecedented access to biological and environmental measurements. Historically, biosystem monitoring relied heavily on hardware sensors, but the limitations in physical accessibility and high cost have prompted a shift toward more sophisticated approaches, paving the way for the rapid development of software sensors capable of addressing these challenges. Concurrently, significant steps have been made in computational methods and techniques for analyzing and interpreting this wealth of biological data.

Monitoring and controlling biosystem variables, such as biomass [5], product concentrations, metabolic rate, and energy expenditure [6], soil organic matter and moisture contents [7,8,9,10], pest outbreak control [11], nutrient-plant management, and greenhouse controlling [12,13,14,15], in real-time or online poses a real challenge. The difficulty stems from issues related to physical accessibility, making certain measurements too difficult. Moreover, the absence of cost-effective and dependable sensors renders some measurements too expensive to undertake. One way to surmount this challenge is to depend on some auxiliary measurable [16,17] variables to indirectly infer the unmeasurable or inaccessible process variables instead of the direct physical measurement of these variables [16]. Such a combination of these auxiliary measured variables (using hardware sensors) and estimation algorithm (software) is termed “software sensor”, soft sensor, inferential sensor, or virtual sensor [16,17,18,19].

This review article explores the innovative application of software sensors for monitoring and controlling biosystems, with a focus on their potential to revolutionize the way we manage complex biological processes. A critical challenge we will address is the mismatch between what is typically measurable and what truly needs to be monitored. This review will highlight how software sensors can bridge this gap by combining hardware sensor data with advanced computational modelling techniques to indirectly infer hard-to-measure target variables, such as stress level, animal and human health indicators, and chemical soil properties. Through three case studies, we demonstrate the advantages of this approach in managing the inherent complexities of biological processes, showcasing its applicability across diverse biosystems. We also address potential challenges and limitations, such as technical hurdles and the need for rigorous calibration against physical measurements. Our aim is to provide a comprehensive overview of software sensors and their potential in contributing to sustainable practices in various sectors, including agriculture, healthcare, and environmental monitoring.

## 2. Model-Based Monitoring and Controlling of Biosystems Concepts

The fourth industrial revolution, also known as Industry 4.0, has brought significant advancements in sensor technologies (e.g., Internet-of-Things (IoT) and smart sensors), which led to a substantial improvement in the monitoring and control of biosystems [20]. This has enabled a more accurate, faster, smaller/compact, and notably cheaper means for data gathering and logging biological and environmental data. The advancements in downsizing has facilitated the creation of micro-sensors, which can be integrated into various environments. The utilization of enhanced materials has facilitated the development of sensors with increased levels of sensitivity and selectivity, allowing them to accurately detect even the most subtle changes in biological signals [20,21]. Moreover, with these cost-effective sensing advancements, deploying multiple sensors in the field and integrating multimodal data to make informed decisions becomes possible. For example, in agriculture, different sensors can gather data about soil conditions, weather, and crop health to optimize crop yields [22,23,24]. In many applications, sensor technologies are used with control systems, which provide continuous feedback of process variables to automate biosystem processes. For instance, in industrial biotechnology, advanced sensors can provide continuous feedback on the bioreactor conditions to the control system, optimizing the production of pharmaceuticals and other bioproducts [25].

With that said, in the context of monitoring biosystems, it is often noticed that what we can typically quantify using conventional hardware sensors may not align with what we truly desire to monitor and study. While hardware sensors can provide invaluable data on various physical, chemical, and physiological parameters, hereafter known as “measured variables”, such as temperature, pH level, heart rate, etc., they often capture a very limited snapshot of the holistic reality of the biosystem. We truly aspire to observe and monitor the nuanced behaviours and the emergent status of these systems, hereafter called “target variables” (e.g., performance, stress, health, wellbeing, …), which transcend the capacity of traditional hardware sensors. Thus, bridging this gap between the measured variables (filed data) and the target variable(s) remains a remarkable challenge to monitor and control many biosystems [16].

Furthermore, in many cases of biosystem applications, it is often that several vital biosystem-related variables are challenging to measure directly using hardware sensors due to their difficulty (e.g., plant biomass, chemical soil properties, plant nutrient dynamics), inaccessibility (e.g., intercellular signalling, pain and thermal perceptions, and ecological relationships in remote or extreme environments), or high cost (e.g., energy expenditure, eDNA, and proteomics).

**Figure 1:**
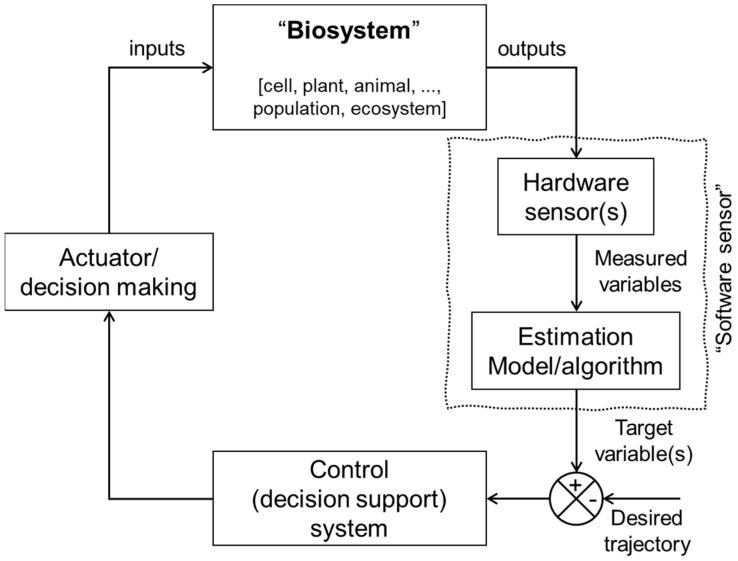
A general schema for model-based monitoring and control (M_b_MC) of biosystems and the employment and software sensor concept. The system is composed of various interconnected components: (a) The biosystem, which encompasses the target biological entity or process being monitored and controlled. (b) Hardware sensors, which collect data on measurable variables. (c) The estimation model, which utilizes computational algorithms to infer target variables (e.g., biomass, soil moisture, animal health status) that are not directly measurable. (d) The control system, which utilizes the estimated target variable data to adjust input variables and optimize the performance of the biosystem. (e) The software sensor, which acts as a virtual representation of the target variable, connecting the measured variables from hardware sensors to the control system.

A model-based monitoring of biosystems is a promising tool for overcoming the aforementioned challenges associated with the direct use of hardware sensors. Instead, it employs the software sensor approach, integrating mathematical models or computational algorithms with sensor data [16]. This approach can offer a more comprehensive and accurate estimation of biosystem target variables. Accordingly, this can enhance our ability to predict, control, and optimize biosystems, ultimately promoting better decision-making in fields such as medical and veterinarian diagnostics, biotechnology, and environmental and agricultural sciences. Figure 1 depicts a general schema of the model-based monitoring and control (M_b_MC) system [16]. The system begins with the biosystem, represented by a block labelled “Biosystem”, which encompasses a range of living components, from individual cells and organisms to entire ecosystems. The biosystem interacts with its surrounding environment and is affected by various inputs, including external factors such as weather conditions and control signals. The outputs of the biosystem, also referred to as bio-responses [1,26], can be directly measured using hardware sensors. The measured variables (e.g., acceleration, video/sound signals, satellite images, and soil temperature), also known as field sensor data, are integrated with the estimation algorithm component (block) to indirectly infer the target variable (e.g., infection, animal stress, growth rate, and soil organic matter), which is the variable directly related to the final objective of the monitoring system [27,28].

The estimated target variable is then used by a control system, which can be a decision support system or an automated control system. This system makes decisions based on the continuously updated data from the target variable, adjusting input variables to optimize the performance [29] of the biosystem (e.g., bio-productivity). For example, the control system could regulate environmental conditions within a greenhouse or adjust feeding schedules for livestock based on real-time estimates of plant and animal growth rate.

The software sensor is a critical component of the M_b_MC system, bridging the gap between the measured variables and the target variables by providing a virtual representation of the target variable. It is this capability that allows for the precise and effective monitoring and control of complex biosystems.

## 3. Software Sensors

The advent of the digital revolution has drawn attention to the need for a different type of sensor capable of performing computational inferences and estimating unreadily observable variables. This has given rise to the concept of software sensors, which are also known as soft, virtual, and inferential sensors. The concept of software sensors is based on the idea of inferring (estimating) essential biosystem variables (target variables) that are challenging to measure directly by fusing data acquired from hardware sensor technology with an estimation model or algorithm (i.e., software). As illustrated in Figure 1, the software sensor comprises two main components: the hardware sensor(s), responsible for generating the measured variable(s), and the software component, which encompasses an estimation algorithm for the target variable prediction.

### 3.1. The Emergence of Software Sensor

The concept of software sensing originated from the domain of systems and control theory [17,30,31]. In many control applications, we need to estimate some internal states of a dynamic system that are unmeasured or difficult to measure for control purposes. The particular estimation algorithm is known as a “state estimator” in the context of control theory, by which the future values of the system states are estimated or predicted based on the current measured states together with the dynamic model of the system [32]. Depending on how uncertainties in a process are treated, state estimation methods can be deterministic or stochastic. The former is usually referred to as “state-observers”, and the latter is called Bayesian filtering, a general statistical method for estimating an unknown probability distribution over time with sensor measurements and a mathematical model. A well-known Bayesian filtering is the Kalman filter, which is tailored for linear dynamic systems and Gaussian noises. For more detailed information on the concepts of state observers and the Kalman filter, please refer to Section 3.2.

**Figure 2:**
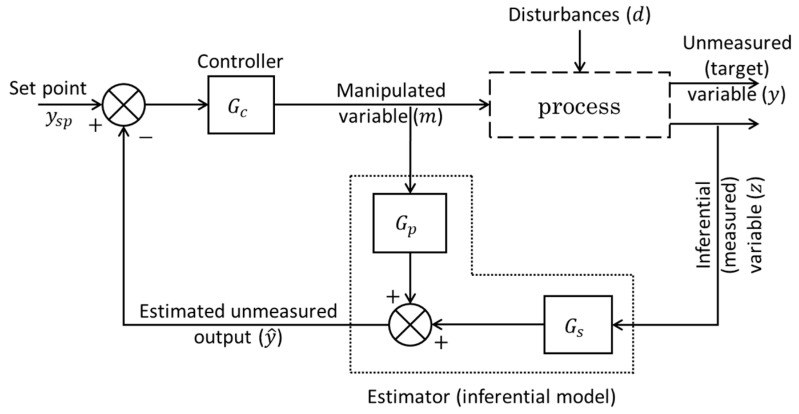
This diagram illustrates the concept of inferential control, based on Brosilow’s work [33], where an unmeasured process variable (y) is estimated using an inferential model. The estimated value is then used by the controller to manipulate the manipulated variable (m) in order to achieve the desired set-point ($y_{sp}$). The system also includes a disturbance (d) that affects the process and an inferential variable (z) that is measured and used to estimate the unmeasured variable.

Additionally, the principle of the software sensor aligns closely with the concept of inferential control proposed in the 1970s by Brosilow [33]. The concept of inferential control is illustrated in Figure 2. This diagram highlights the key components of this approach. In the inferential control scheme (Figure 2), the controlled variable (i.e., the primary process variable, or the target variable, y) is not measured directly but is instead estimated from secondary (inferential) measured variables. These inferential variables (z) are chosen to maintain a strong correlation with the unmeasured output variable (y) in response to changes in the process input (manipulated) variables (m). In this context, the software sensor is represented by the combination of the measured inferential variables and the estimator (inferential model), which, in this specific example [33], is formulated using the transfer functions $G_{p}$ and $G_{s}$ (as shown in Figure 2). Here, the estimated value ($\hat{y}$) of the unmeasured output variable (y) is performing the job of the regular output as if it were measured in the feedback control loop. In essence, the estimator (inferential model) in the inferential control scheme (Figure 2) serves as the software sensor, integrating the measured inferential variables with a model to infer the unmeasured variable.

### 3.2. State Observers and Kalman Filters

**Figure 3:**
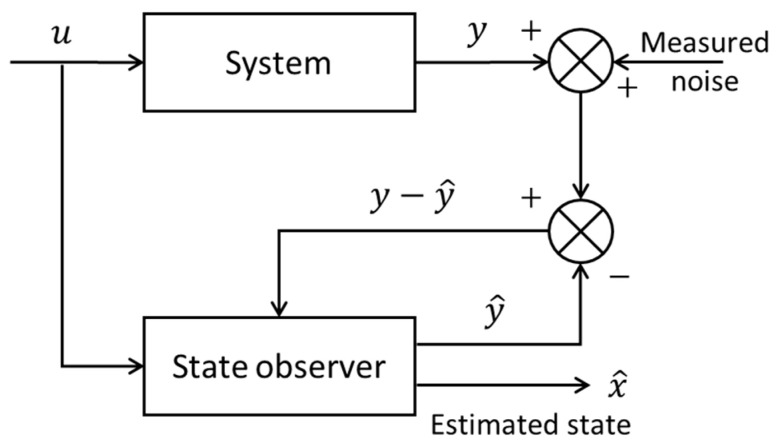
Schematic representation of a state observer integrated within a control system.

State observers and Kalman filter are widely recognized as the earliest and most commonly used forms of software sensors. Their foundational role in this domain stems from their robust capabilities in unmeasured state estimation and extensive applicability across various fields. In the systems and control theory, a dynamic system is often described by an input–output state space model, which is a first-order differential equation about state variables *x* for continuous-time systems or a difference equation about *x* in the discrete-time case. The input variables *u* impart energy or force allowing an alteration of the state variables, and the measurement signals *y* are the system’s outputs. A state observer estimates the internal states *x* of a dynamic system based on available measurements *y* and the input variables *u*. The diagram of the state observer is shown in Figure 3. The state observer utilizes a state space model of the system, incorporating the measurements *y* and the inputs *u*, and by comparing the predicted outputs $\hat{y}$ with the actual measurements *y*, the state observer provides the estimates $\hat{x}$ of the internal states and continuously refines the estimation over time. This iterative process enables the observer to provide real-time or near-real-time estimates of internal states, facilitating feedback control strategies and enhancing the overall performance of control systems in complex and dynamic environments.

The following will look at the commonly employed Luenberger observer as an illustrative example. Consider a discrete-time linear state space model:

(1) x(k+1)=Ax(k)+Bu(k)

where *y*(*k*) = *Cx*(*k*), and *A*, *B*, and *C* are constant matrices. The values of state variables at time k+1, x(k+1) are predicted from current value x(k) and inputs u(k) using the state equation. The second equation then describes how the outputs y(k) are generated. In the Luenberger observer, the difference between the predicted output $\hat{y}(k)$ will be subtracted from the measured outputs y(k) and then amplified by a constant gain matrix L to correct and refine the estimates of state variables, as described by the equations below.

(2) $$\hat{x}\left(k+1)=A\hat{x}(k\right)+Bu(k)+L[y(k)-\hat{y}(k)]$$

where $\hat{y}(k)$ = *C*$\hat{x}(k)$.

Provided a system is observable, namely, the so-called observability matrix

(3) $$O=\left[\begin{array}{c} C\\ \begin{array}{c} CA\\ \vdots \end{array}\\ {CA}^{n-1} \end{array}\right]$$

is full rank, the matrix parameter L can be designed such that the observer error $e(k)≔\hat{x}(k)-x(k)$ converges to zero as *k* goes to infinity. More precisely, the Luenberger observer error follows the dynamics $e\left(k+1\right)=\left(A-LC\right)\;e(k)$, and therefore, when A−LC is stable (i.e., all its eigenvalues are inside the unit circle), then the error dynamics is asymptotically stable, meaning that e(k) converges to zero as k increases, for any initial error e(0).

The above Luenberger observer is a type of full-order observer that aims to estimate all the state variables. On the other hand, reduced-order observers are an alternative approach for software sensor that uses system measurements to estimate only the ‘hidden’ states (i.e., target variables) as a subset of the state variables of a system. While reduced-order observers may be more challenging to design compared with their full-order counterparts, they offer advantages in terms of computational efficiency, simplicity, and potential for improved performance in certain applications [34,35,36].

The Kalman filter is named after Rudolf E. Kálmán (19 May 1930–2 July 2016), who published his famous paper [37] that describes a recursive method for estimating the state of a linear dynamic system from a series of measurements with Gaussian noises. Consider the linear discrete-time system:

(4) $$x_{k}={Ax}_{k-1}+{Bu}_{k}+w_{k}$$

(5) $$y_{k}={Cx}_{k-1}+v_{k}$$

where $x_{k-1}$ denotes the state at time k−1, and $x_{k}$, $u_{k-1}$, and $y_{k-1}$ are the state, control input, and measured output at time k, respectively. Moreover, the constant matrices A, B, and C are referred to as transition matrix, input matrix, and output matrix, respectively, $w_{k}$ is the Gaussian process noise with covariance matrix Q, and $v_{k}$ is the measurement noise with covariance matrix R. The objective of the Kalman filter is to discover the probability distribution of the state x at time k.

**Figure 4:**
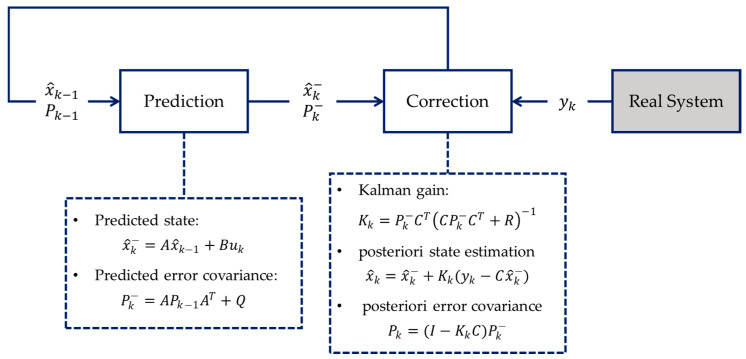
Flowchart illustrating the two-step process of the Kalman filter algorithm for state estimation in a linear discrete-time system. In the prediction step, the state and error covariance are projected one step ahead, and then in the correction step, using the measurement data from a real system, the estimated state and covariance matrix are updated. These updated values are fed into the next prediction cycle.

The standard Kalman filter is a two-step process, as shown in Figure 4. The first step is prediction, i.e., to compute the state prediction and the error covariance P based on the linear model without sensor measurements. This error covariance matrix P can be considered a measure of uncertainty in the estimated state. This variance comes from the process noise and propagation of the uncertain ${\hat{x}}_{k}^{-}$. At the start of the algorithm, the values for $\hat{x}$ and P come from their initial estimates. The second step is called *correction*, where we weigh in the sensor measurement. More precisely, the a priori estimates calculated in the prediction step are updated to find the a posteriori estimates of the state ${\hat{x}}_{k}$ and error covariance $P_{k}$. To do so, the Kalman gain *K* is calculated such that it minimizes this new error covariance $P_{k}$, and it determines how heavily the measurement and the a priori estimate contribute to the calculation of $x_{k}$, or simply speaking, how much the sensor measurements can be trusted. If we have sensors providing accurate measurements, meaning that the measurement noise is small, then the measurement can be trusted more and have more weight such that it contributes to the calculation of $x_{k}$, more than the a priori state estimate does. In the opposite case where we have bad sensors, the measurement noise will be large, namely a large R, then the sensors are trusted less, so in the equation, the computation of $P_{k}$ mostly comes from the a priori estimate.

Aside from the standard Kalman filter, which is widely used for state estimation in linear dynamic systems with Gaussian noise, there have been various developments and variations to accommodate different types of systems and noise characteristics. Below, we list some of the notable ones:

(1) Extended Kalman Filter (EKF) [38,39]: This is an extension of the Kalman filter to nonlinear systems. It linearizes the nonlinear system at each time step around the current mean and covariance estimate and then applies the standard Kalman filter equations. While widely used, the EKF has limitations, especially when dealing with highly nonlinear systems or when linearization errors are significant.
(2) Unscented Kalman Filter (UKF) [40]: The UKF addresses some of the limitations of the EKF by approximating the mean and covariance through a set of carefully chosen sample points (called sigma points) rather than linearization. It captures the mean and covariance of the state distribution more accurately in nonlinear systems and is more robust to nonlinearity than the EKF.
(3) Ensemble Kalman Filter (EnKF) [41,42]: The EnKF is an extension to handle large-scale systems, which uses a Monte Carlo approach with a finite number of ensemble members. Essentially, it makes use of sample covariance in place of the traditional covariance matrix.
(4) Particle Filter [43] is a non-parametric filter representing the state estimate as a set of weighted particles (similar to the ensemble members in EnKF). Unlike other Kalman filter variants, the particle filter does not rely on Gaussian assumptions, and therefore, it can deal with highly nonlinear and non-Gaussian systems. However, this flexibility comes at the cost of efficiency compared with EnKF.

### 3.3. Software Sensor Design

This section provides a brief overview of the common methodologies for designing software sensors, aiming to help non-expert readers understand the main components of the design process without delving into exhaustive theoretical and mathematical details. For more in-depth theoretical insights, readers are encouraged to refer to the cited bibliography.

The model, or estimation algorithm, is the cornerstone of the software sensor (refer to Figure 1), making its development and identification the most critical step of the software sensor design process. Hence, based on the modelling approach employed in the estimation algorithms, software sensors can be classified into two subtypes: the hypothetico-deductive (or white-box) approach and the inductive (or black-box) approach. In the following subsection the main differences in both modelling approaches are explained.

#### 3.3.1. Modelling Approaches

The hypothetico-deductive (white-box) modelling approach involves defining a priori conceptual model structures based on first principles (ab initio) assumptions derived from established scientific paradigms [44]. This method, also known as mechanistic modelling, relies on fundamental physical laws such as thermodynamics, fluid dynamics, and heat transfer. For example, the calculation of food thermal properties for designing storage and refrigeration equipment strictly using heat transfer and thermodynamics laws, without incorporating any empirical models or fitting parameters, exemplifies a deductive modelling approach. The primary advantage of this method is its ability to provide a deep mechanistic/physical understanding of the process, leading to high accuracy if the system is well understood and the necessary parameters are available. However, this approach often requires extensive domain knowledge and detailed data, which can be a limitation in complex or poorly characterized systems.

The inductive or data-driven (black-box) modelling, on the other hand, abstains from making theoretical preconceptions at the early stages of analysis [44]. Instead, it aims to discover patterns directly from the observational data. The model structure in this data-driven approach is not pre-specified but is inferred from the data using statistical and machine learning techniques. Inductive modelling encompasses a variety of methods, including artificial neural networks (ANN), support vector machines (SVM), and more recently, advanced deep learning techniques. These methods excel in capturing complex patterns and relationships within large datasets without requiring detailed knowledge of the underlying physical/physiological processes. The rapid growth in computational power and data availability has significantly expanded the use of inductive modelling in soft-sensing applications. However, the accuracy of these models is highly dependent on the quality and quantity of data, and they may struggle to generalize beyond the conditions represented in the training data.

The choice between deductive and inductive approaches depends on several factors, including the complexity of the system, the availability of process data, the level of understanding of the underlying process mechanisms, and the specific objective of the software sensor design.

Hypothetico-deductive reasoning has long been integral to biological research practice, underpinning foundational theories such as evolution and germ theories. However, the inherent complexity and nonlinearity of biological systems make extreme deductive (pure white-box) modelling approaches challenging [1,16]. This often results in highly intricate mechanistic models characterized by numerous differential equations and parameters. For instance, metabolic processes and pathways are typically modelled using a series of differential equations that describe the time evolution of metabolites and enzyme concentrations in cellular biochemical reactions [45]. Such computational complexity can place a significant burden on resources, leading to slower convergence times. This, in turn, hampers the practical application of these models in real-time or online software sensing applications.

Conversely, the inductive approach prioritizes practical problem-solving irrespective of the method used. While an extremely inductive (data-driven) model might accurately fit the available data, it can lead to false predictions without understanding the underlying system and mechanisms.

Nevertheless, the inductive approach offers a more pragmatic solution for designing software sensors, especially when the primary goal is to achieve practical and timely results without needing intricate mechanistic details. Additionally, this approach leverages the power of empirical data and advanced computational techniques to develop models that can adapt and respond to real-time data with greater flexibility and efficiency. Therefore, for the sake of convenience, this article focuses exclusively on data-driven software sensors.

#### 3.3.2. Data-Driven Software Sensors

Designing a software sensor using a data-driven approach involves several critical steps to infer the unmeasured (target) variable from measured (auxiliary) data. These steps encompass data acquisition, the selection of auxiliary variables and features, modelling, model validation and testing, and implementation. In this article, we will mainly focus on the modelling step of the design process, including the selection of the appropriate data-driven modelling technique. For more comprehensive information on the other essential steps, readers can consult specific literature sources such as [17,18,46,47,48,49], which provide in-depth discussions and methodologies for effective data acquisition and preparation, feature engineering, and system integration needed for the software sensor design process.

**Figure 5:**
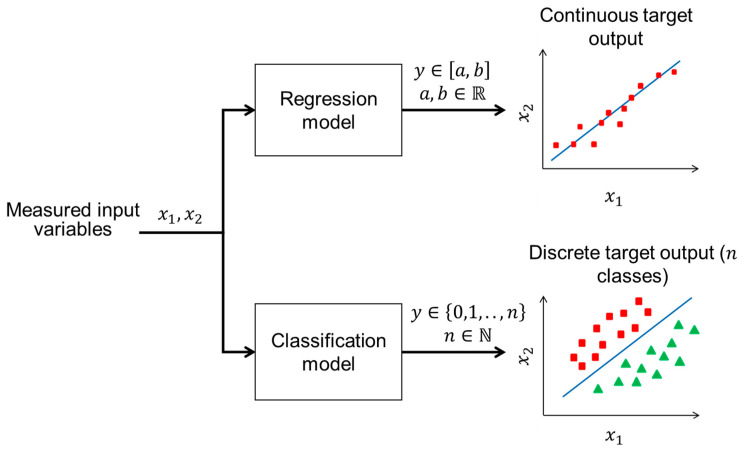
This diagram illustrates the fundamental difference between regression and classification models. In regression, the goal is to predict a continuous target variable (e.g., animal weight, soil organic matter level) represented by a real number within a specified range. In classification, the objective is to predict a discrete target variable (e.g., health vs. sick animal) represented by a category or class.

The choice of data-driven modelling techniques is fundamentally driven by the nature of the target variable that needs to be estimated. When the target variable is continuous, meaning it can take any numerical value y within a range [a,b]∈R, a regression model (Figure 5) is typically employed. Regression models are supervised learning algorithms designed to predict continuous value by identifying the relationship between measured input (auxiliary) variables and the continuous target output. For instance, predicting cell growth rate [50], animal weight [51] or biomass concentration [45] in biosystem monitoring or control applications would necessitate the use of regression techniques such as linear and polynomial regression [52], decision trees for regression [53], or more advanced methods such as support vector regression (SVR) [54] or neural networks (NNs) [55].

On the other hand, when the target variable is discrete, meaning it can only take on a specific set of discrete values or labels $y\in \left\{0,1,\ldots ,n\right\},\;n\in N$, a classification model is the appropriate choice. Classification models are used to predict categorical outcomes by learning from the patterns and relationships in the input data. For example, determining the presence or absence of a particular condition in biosystems, such as infection status [56,57], or predicting different levels of biological responses, such as thermal sensation [58], would require the use of classification techniques such as logistic regression [59], k-nearest neighbours [60], decision trees and random forests (RF) [61], support vector machines (SVM) [60], or NNs [62].

## 4. Applications and Biosystem Case Studies

Software sensors are utilized in many applications across diverse fields, spanning from manufacturing, industrial processes, environmental engineering, and energy management to medical applications, agriculture, biology, and beyond. Table 1 presents an overview of exemplary applications of the software sensing approach in different fields.

The utilization of software sensors in the fields of biology and biosystems is highly prevalent. In digital agriculture, for instance, software sensors emerge as game-changers. Traditionally, estimating soil moisture content, for example, relied on costly methods such as wireless sensor networks and satellite imagery, which often lacked the required precision [63]. Researchers have introduced an innovative solution to these challenges—a soft sensing algorithm leveraging deep learning techniques [7]. This approach creates a virtual soil moisture sensor, bypassing the limitations associated with physical sensors and offering a more accurate and cost-effective solution. The integration of software sensors has similarly revolutionized practices in animal sciences and precision livestock farming (PLF), offering non-invasive means (Table 1) of monitoring animal behaviour, health, and welfare in real-time. For example, Youssef et al. [64,65] employed a software sensing approach to non-invasively monitor the cardiogenic signal and heart rate from moving pigs and incubated avian embryos.

Moreover, advancements in computer vision algorithms and imaging technology enable the real-time monitoring and recognition of animal activities [66,67,68]. Additionally, software sensors are employed in early warning systems, such as in [56,69,70,71], for infection and animal health problems using contactless sensors (e.g., microphones). With the advancements in software sensor technology, digital healthcare is being promoted to change people’s lives [72]. Data reliability and robustness can be enhanced by constructing sensor arrays that simultaneously gather comprehensive biological parameter data from multiple body locations [72].

**Table 1:** Overview of software sensor applications in different fields.

	Field	Example Applications
Various applications	Manufacturing and industrial processes	- Process Industry: [48,73]
	Environmental engineering	- Wastewater management: [74,75,76] - Environmental monitoring: [77] - Ecology: [78]
	Transportation and smart cities	- Smart cities: [79,80,81,82]
	Cybersecurity	- [83,84,85,86,87,88]
Biosystems applications	Biology	- Neuroscience: [72] - Molecular and cell biology: [50,89,90] - Fermentation: [50,83,89,90,91] - Bioprocess monitoring and control: [47,50,92,93,94]
	Medical and human health	- Digital Healthcare: [52,95,96,97,98] - Pharmaceutical and drug discovery: [99,100]
	Agriculture and animal health	- Digital agriculture: [101,102] - Soil moisture estimation: [7,103,104,105] - Greenhouse monitoring and control: [106,107]
		- Precision livestock farming (PLF): [51,64,65,66,68,108,109,110,111]

While this review focuses on the application of software sensors in biosystems, it builds upon previous research works in the field of model-based monitoring and the control of different biosystems, such as [1,69,112,113,114,115,116,117]. This review extends these previous efforts by focusing on the unique challenges of biosystems and exploring the potential of software sensors in managing their inherent complexity.

In the following subsections, we present three case studies to illustrate the applications of data-driven software sensors in monitoring and controlling various biosystems. The first case study demonstrates the use of software sensors for the real-time monitoring of animal health, specifically as an early warning tool for respiratory infections. The second case study showcases the application of software sensors in the fusion of different sensor data to estimate difficult-to-measure biosystem variables, such as soil organic matter. Lastly, the third case study explores the use of software sensors for the automatic control of biosystems, exemplified by a greenhouse environment.

### 4.1. Early Warning System: Software Sensor for Real-Time Monitoring off Animal Respiratory Infection

In general, respiratory infections pose a significant challenge to animal health and welfare, especially in the case of intensive animal farming, particularly among calves and pigs. The increased severity and mortality rates associated with such infections show the pressing need for a robust early warning system. In current practice, the reliance on manual illness detection and subsequent veterinarian intervention seems time-consuming, does not occur early enough, and is expensive [69]. Traditionally, respiratory infections and sounds in commercial livestock farms are typically monitored via direct human observation [118]. However, as herd sizes grow and production intensifies, individual animals receive less attention [119]. This decline in available time and resources may cause producers to overlook signs of respiratory diseases [118,120], often resulting in late intervention when symptoms become noticeable. Hence, there is a compelling call for an efficient automatic monitoring system to enhance the early detection of animal respiratory infections and their related outcomes, such as mortality. A comprehensive review of various automated monitoring techniques for animal respiration and sounds, highlighting their advantages over traditional methods, is provided in [118].

Automatic monitoring systems should be robust, providing continuous and unobtrusive surveillance without disrupting the animal’s environment. Additionally, detecting the infection at an early stage requires swift observation of the clinical signs associated with the disease.

**Figure 6:**
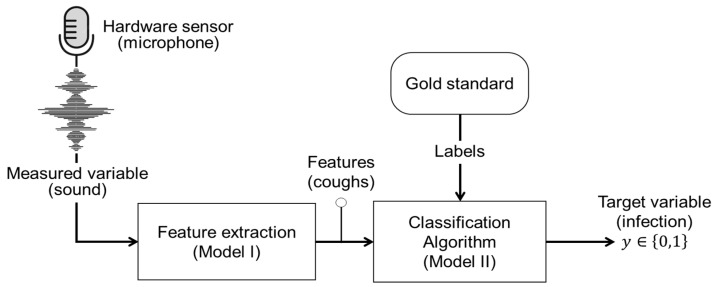
Schematic representation of a software sensor designed for early warning of animal respiratory infection.

Coughs are one of the primal clinical signs associated with respiratory infections such as bovine respiratory diseases (BRD) [57]. The distinctive sound of coughing can serve as a key feature indicator for predicting respiratory infections in animals. The advantage of coughing sounds, and bioacoustics in general, lies in their ability to be measured noninvasively and remotely using microphones, ensuring minimal disruption to the animal’s normal behaviour [56]. Over the past two decades, different research efforts (e.g., [56,69,112,121,122,123,124,125,126,127]) have utilized sound measurements in conjunction with various algorithms to extract acoustic features indicative of respiratory infections. This approach can essentially be perceived as a software sensor, operating to indirectly predict an animal’s infection status. In this section, we will clarify the primary structure and main components (Figure 6) of the software sensor employed to predict whether an animal is infected based on its coughing sound. As illustrated in Figure 6, the target variable in this context is a binary label, $y\in \left\{0,1\right\}$, indicating infected = 1 or not-infected = 0 animal status. The measured variable is sound signals recorded using microphones, representing the hardware sensor component, see Figure 6. Functioning as the software component, two algorithms, namely, Model I and Model II (see Figure 6), are utilized as follows:

#### 4.1.1. Model I: Feature Extraction and Coughing Sound Recognition

This algorithm is responsible for extracting the key feature indicators, the cough sound, and other features from the measured variable—specifically, the sound signals—associated with the animal respiratory infection.

**Figure 7:**
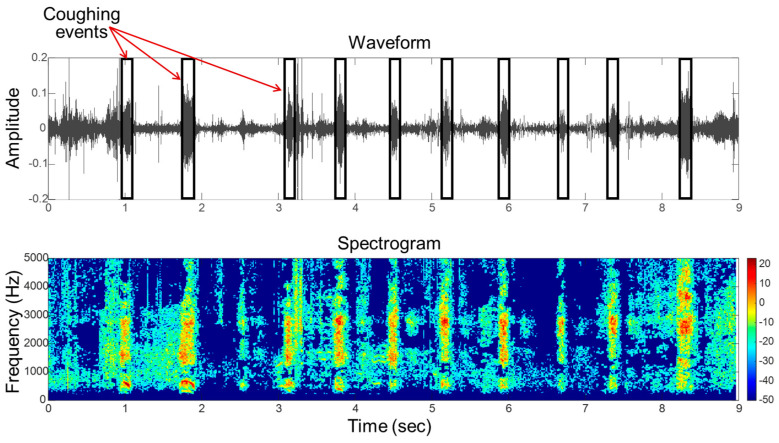
Exemplar waveform (**upper graph**) and spectrogram (**lower graph**) of the coughing sound acquired from a sick pig with pneumonia.

For coughing sound recognition, acoustic features can be manually extracted from the audio waveforms. Specifically, the Mel-frequency cepstral coefficient (MFCC) has been used for pig cough recognition and classification [70]. Moreover, other time and frequency domain features, such as power spectral density (PSD), spectral entropy, and root mean squares (RMS), are frequently used for cough sound recognition in both pigs and calves, as evidenced in studies [56,69,128]. An exemplar waveform and spectrogram visualization [113] of extracted coughing sounds from sick pigs is depicted in Figure 7. The presented coughing sound data are recorded during an experimental study; for more information, see [129].

Alternative approaches utilize machine learning techniques, such as CNN, for coughing sound classification and recognition. For instance, Yin et al. [71] employed a deep CNN model (AlexNet) in an end-to-end manner to recognize sick pigs’ coughs. However, this approach is computationally demanding and impractical for real-time applications. To overcome these drawbacks, researchers like Shen et al. [122] adopted a pragmatic strategy by employing shallow CNN to extract deep acoustic features. These features were then fed to a lighter classifier, such as SVM, to recognize pigs’ coughing sounds. An extensive review of cough sound recognition and classification approaches is represented by Legua et al. [130].

#### 4.1.2. Model II: Detection of Infected Animals

This algorithm, which is a supervised classification model in this context, learns from the labelled dataset, using the gold standard, to assign the extracted input features (e.g., number of coughing events), using Model I, to one of the target variable labels. In the context of this example, the target variable poses only two possible labels, namely infected or not-infected. Thus, the goal of Model II is to solve the classification problem y=F(x), where F(⋅) is a decision function that maps the input features x to the output (target) variable y.

To find the optimal parameters for F(⋅), the model needs to be trained on a given labelled dataset. Consider the labelled training set ${\left\{\left(x_{i},y_{i}\right)\right\}}_{i=1}^{n}$ with input data $x\in R^{n}$, where n∈N is the number training samples, and output data $y_{i}\in R$ with class labels $y_{i}\in \left\{0,1\right\}$ indicating infected (1) or not-infected (0). The classification problem presented here can be solved by a binary classifier such as SVM, NNs, random forest, logistic regression, etc. Binary classifiers involve finding the parameters that minimize a certain cost or loss function. The specific form of the function F and the associated optimization process depends on the chosen algorithm. For example, in logistic regression, a sigmoid function is defined as follows:

(6) $$F\left(x\right)=\frac{1}{1+e^{-(\beta_{0}+\beta_{1}x_{1}+\ldots +\beta_{k}x_{k})}}$$

where $\beta_{0},\;\beta_{1},\ldots ,\beta_{k}$ are the parameters to be optimized during the training process to find a decision boundary that separates the two classes in the defined feature space ${\langle x_{1},\ldots ,x_{k}\rangle \in R}^{k}$.

### 4.2. Sensor Fusion: Software Sensor Application for Indirect Estimation of Soil Organic Matter

Assessing soil organic carbon (SOC) is a vital indication of soil health due to its importance in carbon sequestration, fertility, and soil moisture content [131,132]. Precisely mapping SOC levels in various landscapes helps evaluate ecosystem benefits, direct sustainable land-use methods, and reduce climate change impacts [133,134]. Also, advanced sensing technologies and data analytics now have a vital function in sustainable biosystem management, whether it involves optimizing crop growth conditions in high-tech greenhouses or evaluating the carbon storage potential of soils at various scales [133,135,136]. Both arenas highlight the significance of accurate, data-driven methods for protecting the health and productivity of key biological systems on the planet, regardless of their various scales and properties.

**Figure 8:**
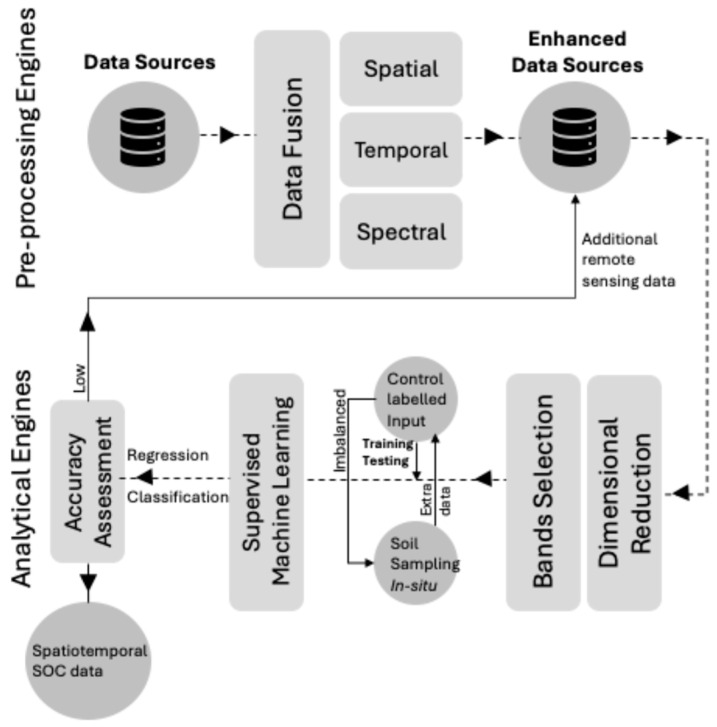
Software sensor process diagram for soil organic carbon (SOC) prediction. The diagram illustrates the integration of remote sensing data with supervised machine learning algorithms to estimate SOC levels. The process begins with data fusion of spatial, temporal, and spectral data sources, including satellite images and field measurements. These fused data are then processed through supervised machine learning techniques, with labeled inputs from in situ soil sampling to calibrate the model, band selection, and dimensionality reduction to optimize the spectral inputs for SOC prediction. The final step assesses the model’s accuracy using spatiotemporal SOC data, allowing for robust predictions.

The use of hyperspectral remote sensing technology is a significant advancement in digital agriculture due to its ability to capture soil surfaces with exceptional spectral precision that can reach hundreds of spectral bands (i.e., groups of a particular range of wavelengths) [137,138,139]. Hyperspectral imaging using the visible near-infrared (VIS-NIR) is particularly effective in detecting near-real-time fine spectral differences, as each material on the soil’s surface has a unique spectral signature akin to a fingerprint, which is often called “spectral signature”. This allows sensing capability to discriminate between different soil surface materials and improve SOC predictions [140,141,142,143,144]. In this case study, a software sensor is employed to estimate SOC. Such application requires following specific data management and processing frameworks such as those shown in Figure 8.

Software sensors begin their role at the data fusion stage, where hyperspectral imaging data are combined with other relevant data sources such as climate data, soil type, and historical crop yield data. Spectral features that correlate with SOC levels are extracted using methods like principal component analysis (PCA) or selecting spectral indices sensitive to SOC. This approach reduces dimensionality while preserving variance in spectral data [145,146]. PCA is a technique that converts the initial spectral data into a collection of linearly independent variables known as principal components (PC) and are given as follows:

(7) $$PC\;\left(X\right)=\;W^{T}\cdot \;X$$

where *X* is the matrix of spectral data and *W* is the matrix of weights or coefficients. Once relevant spectral features are extracted, two main modelling approaches might be considered; linear regression can be used when there is low spatial heterogeneity between the observation [147].

(8) $$Y=\beta_{0}+\beta_{1}\cdot x_{1}+\beta_{2}\cdot x_{2}+\ldots +\beta_{n}\cdot x_{n}+\varepsilon ,$$

where Y is SOC content; $X_{1},\;X_{2},\ldots ,X_{n}$ are the spectral features, $\beta_{0},\;\beta_{1},\;\beta_{2},\;\ldots ,\;\beta_{n}$ are the coefficients that describe the impact of each spectral feature on the SOC, and ε is the error.

For more complex relationships, nonlinear models or machine learning techniques such as the SVM, RF, or NNs might be employed. These techniques can more effectively capture nonlinearities and interactions between spectral features [148,149].

SVM models are utilized for predicting soil organic carbon (SOC) levels, whether through spatial classification or regression [150]. The SVM algorithm works by identifying an optimal hyperplane that best separates different groups of SOC data points in a high-dimensional space, thus enabling accurate the categorization and prediction of SOC levels [151].

The SVM model for SOC classification can be represented as follows:

(9) $$f\left(x\right)=w\cdot \emptyset \left(x\right)\;+\;b,$$

where x is the input feature vector, $\emptyset \left(w\right)$ is the feature vector transformed into a high-dimensional space, w is the weight vector, and b is the bias term. The decision function is based on the sign of $f\left(x\right)$.

RF is a machine-learning technique used for classification, regression, and other tasks. It works by creating many decision trees during the training process. A simple representation for an [152,153] RF regression model is the average of all the decision trees:

(10) $$f\left(x\right)=\frac{1}{N}\sum_{i=1}^{N}f_{i}\left(x\right),$$

where $f_{i}\left(x\right)$ is SOC prediction of the i-th decision tree, and N is the number of trees in the RF.

Neural networks, especially deep learning models, are capable of capturing complex non-linear relationships. A simple feedforward neural network for regression can be mathematically represented as a series of transformations:

(11) $$\begin{array}{c} h_{1}=\sigma \left(W_{1}x_{+}b_{1}\right)\\ h_{2}=\sigma \left(W_{2}h_{1}+b_{2}\right)\\ \begin{array}{c} \vdots \\ f\left(x\right)=W_{k}h_{k-1}+b_{k} \end{array} \end{array},$$

where x is the input, $h_{i}$ are the hidden layers, $W_{i}$ and $b_{i}$ are the weights and biases of the i-th layer, and σ is a non-linear activation function such as the sigmoid, tanh, or ReLU. For capturing non-linearities and interactions between spectral features, the convolutional neural network (CNN) can be used when (e.g., raster data) derived from hyperspectral space-borne sensors such as the Environmental Mapping and Analysis Program (EnMAP) and the Preferred Reporting Items for Systematic Reviews and Meta-Analyses (PRISMA), or a Recurrent Neural Network (RNN) if the data are sequential, which depends on being multi-temporal, such as in the Open Soil Spectral Library (OSSL) [135,154,155,156,157].

### 4.3. Biosystem Control: Software Sensor Application for Automated Greenhouse Control

The modern greenhouse is a complex dynamic system that offers a controlled or partially enclosed space for plants, shielding them from external weather fluctuations. To foster an environment suitable for growing crops while minimizing energy usage, efficient control and optimization strategies have been applied to regulate greenhouse climate conditions, e.g., [13,158,159]. However, implementing these advanced control strategies is hardly achievable if we do not have accurate information about climate conditions and crop growth status. It is reported by [160] that sensor errors can lead to a significant negative effect on crop yield and energy use in the greenhouse.

In practice, the sensors that measure the climate states, such as air temperature, humidity, and carbon dioxide level, can be affected by noises from various sources. Moreover, the real-time and non-invasive measurement of the crop growth states, such as leaf area, dry weight, and fruit content, presents an even greater challenge due to the complex nature of plant physiology. In this context, soft sensing techniques offer promising avenues for addressing these challenges. One such solution is to apply data assimilation through Kalman filtering, and some relevant results can be found in [106,161,162].

**Figure 9:**
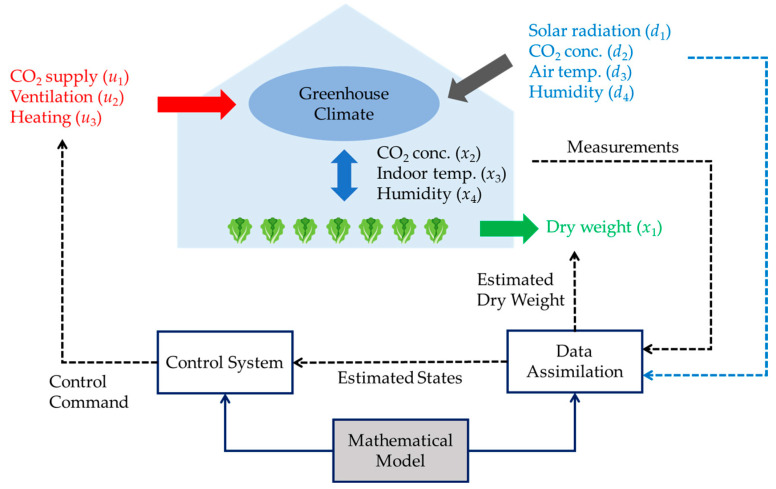
Schematic representation of the lettuce greenhouse model. The system takes real-time measurements of indoor climate variables and estimates the dry weight of the plants through data assimilation, which is updated by a mathematical model. The estimated states are then fed into the control system to adjust inputs and maintain optimal conditions for plant growth.

A typical implementation of data assimilation in the lettuce greenhouse is outlined in [162], where the greenhouse system has a schematic representation, as shown in Figure 9. The microclimate within the greenhouse is characterized by three key variables: CO_2_ concentration *x*_2_, indoor temperature *x*_3_, and humidity *x*_4_. These environmental factors are subject to the influence of external weather conditions (denoted as d), such as solar radiation, external CO_2_ levels, air temperature, and humidity, along with control operations (denoted as u), including CO_2_ supply, ventilation, and heating. The environment states *x*_2_, *x*_3_, and *x*_4_, weather conditions d, and control signal-dedicated sensors with inherent noises measure u. On the other hand, the dry weight of crops *x*_4_ is hard to measure directly in practice. Therefore, the problem of interest here is to filter out the measurement noises, estimate the three environmental states of greenhouses, and infer unmeasurable dry weight using data assimilation.

The data assimilation requires a dynamic model of the greenhouse system in

(12) $$x(k+1)=f\left(x\left(k\right),u\left(k\right),d\left(k\right),w(k))\right)\;y(k)=g\left(x\left(k\right),v(k)\right)$$

with all the variables at time step *k*, namely, state x(k), measured output y(k), controllable input u(k), weather disturbance d(k), process noise w(k), and measurement noise v(k). The variable x(k+1) is the prediction environmental variable at time step k+1. Thereby, the nonlinear function f(·) is a complex function describing how the environmental variables (CO_2_ concentration *x*_2_, temperature *x*_3_, and humidity *x*_4_) and the crop variable (dry weight *x*_1_) change over time, given the external weather condition d and control inputs *u*. In [162], the ensemble Kalman filter is employed for data assimilation, which roughly contains two main steps:

- Step 1: Predict the ensemble forward in time using the mathematical model, which simulates the effects of the external weather conditions and the control operations on the greenhouse system.

- Step 2: Update the ensemble with new sensor measurements using a formula incorporating measurement errors and the ensemble covariance. This step adjusts the ensemble to be consistent with the data and reduces the uncertainty of the state estimate. In this step, the algorithm also provides an estimate of the unmeasurable dry weight by including it in the state vector and updating it with the observations of the other variables, using the correlations between them. In [162], the ensemble Kalman filter estimates the parameters in the mathematical mode by treating them as part of the ensemble.

The output of the data assimilation is the estimated state variables, which include an estimation of crop dry weight and the filtered measurement of CO_2_ concentration, indoor temperature, and humidity. These estimations are then passed to control design/decision-making, generating commands to regulate the greenhouse’s CO_2_ supply, heating, and ventilation systems, see Figure 9.

## 5. Considerations for Designing Biosystem Software Sensors

The applications and case studies highlighted earlier have demonstrated the valuable advantages that software sensors can offer in the field of real-time monitoring and the control of biosystems. By combining data from traditional sensors with advanced computational models, these software sensors allow us to indirectly infer crucial variables that are difficult or impossible to measure directly, such as stress levels, animal health indicators, and soil properties. This opens up a wide range of practical possibilities. This enables real-time monitoring and decision-making, leading to more efficient and sustainable practices. For instance, software sensors can provide early warning systems for animal diseases (as shown in case study 1), optimize fertilizer applications in agriculture, and automatically control greenhouse environments for optimal crop growth (as shown in case study 2). These applications not only enhance productivity but also contribute to improved animal welfare, reduced environmental impact, and greater resource efficiency. As sensor technologies and computational capabilities continue to advance, software sensors are poised to play an increasingly vital role in managing complex biosystems for a more sustainable future.

That being said, while software sensors hold great promise, their design and implementation require careful consideration of several key challenges and constraints. In this section, we will address number of common considerations that software sensor designers need to carefully account for, covering challenges related to the inherent complexity of biological systems themselves and the nuances of data-driven modelling approaches.

### 5.1. Challenges Related to the Biological System

Biological systems present a unique set of challenges due to their inherent complexity, non-linear behaviour, time-varying dynamics, and individual variability [1]. These characteristics make it challenging to accurately model and predict the behaviour of biological processes.

#### 5.1.1. Complexity

The complexity inherent in biological systems stems from their multidimensional nature, characterized by intricate interactions between various processes and agents operating at different scales. As a result, biological systems exist on the “edge of chaos”, where they exhibit a delicate balance between order and disorder [163]. While they can show regular and predictable behaviour, these systems are also prone to sudden, massive, and stochastic changes in response to seemingly minor perturbations. This characteristic makes them exceptionally challenging to model, monitor, and control, especially when compared with more predictable man-made systems, such as car engines.

Therefore, when designing a software sensor for monitoring or controlling such systems, it is crucial to select and train data-driven models capable of incorporating stochastic elements to accurately capture and predict the likelihood of sudden changes. For instance, Bayesian networks can be used to predict the probability of sudden changes in disease progression [164] or ecological shifts [165] by modelling the dependencies between various biological factors. Statistical models such as hidden Markov models can be employed in software sensors to detect sudden changes in physiological signals, such as heart rate variability or neural activity patterns [166]. For more complex applications, deep learning models with dropout regularization can be used for image classifications in medical and diagnostic applications [167].

#### 5.1.2. Time Variability

Biological processes are inherently dynamic, characterized by continuous change over time due to numerous internal factors, such as time-dependent biochemical pathways, circadian rhythms, hormonal cycles, and aging, as well as external factors, such as fluctuations in environmental conditions and stressors like pathogens or injuries. This temporal variability poses significant challenges in monitoring, modelling, and controlling biological systems as well. To account for such temporal variations during software sensor design process, continuous and automated data acquisition systems, such as wearable sensors [52], contactless sensors [168,169], and remote sensing technologies [170], can be employed to ensure that critical information and dynamics are not missed. Also, machine learning techniques such as recurrent neural networks, including long short-term memory networks (LSTM), are specifically designed to handle sequential data and can effectively model temporal dependencies in biological systems [171]. For controlling biological systems, adaptive control strategies, such as model predictive controllers (MPC), can anticipate future states and make proactive adjustments to control strategies to accommodate for the time-varying nature of the controlled biological system [50].

#### 5.1.3. Individual Variability

Biological systems are intrinsically characterized by significant individual variability, which adds an extra layer of complexity to their monitoring, modelling, and control. This variability arises from genetic and environmental factors, resulting in diverse responses among different individual of the same species, and even within the same individual at different times [1]. During the modelling process, capturing the full range of responses within a population requires models that can handle high levels of heterogeneity. In such cases, a single, general model often is not enough. Additionally, accurately estimating model parameters becomes challenging when dealing with individual variability, as it requires extensive data from multiple individuals or time points. Therefore, personalized modelling approaches, which can be tailored to specific individuals, can be employed to account for unique individual variability and dynamic responses. For example, a personalized SVM model in combination with k-nearest neighbor (KNN) is utilized to predict individual human thermal comfort [60].

### 5.2. Challenges Related to the Modelling Step

Despite its several advantages, data-driven modelling comes with several challenges that must be addressed to ensure its effectiveness and reliability for software sensor applications. Here are some key challenges:

#### 5.2.1. Model Complexity

**Figure 10:**
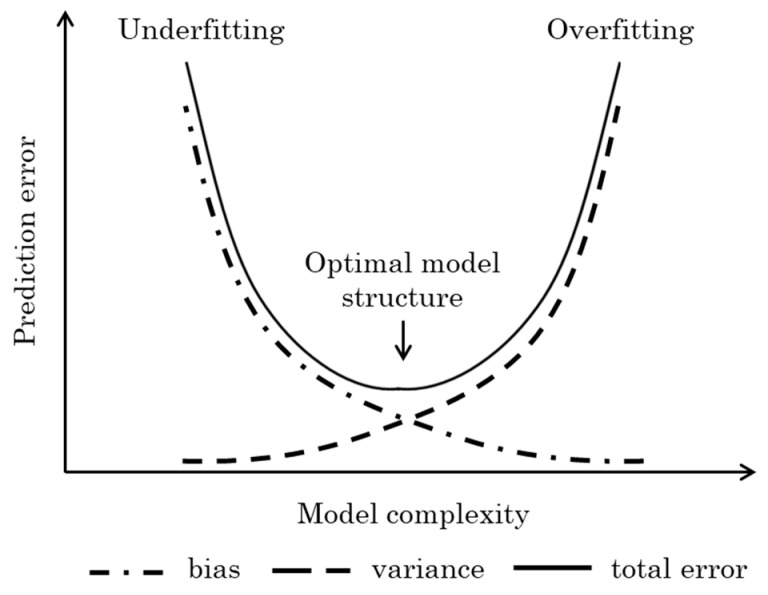
This graph illustrates the relationship between model complexity and prediction error. Underfitting occurs when a model is too simple to capture the underlying patterns in the data, resulting in high bias. Overfitting, on the other hand, happens when a model is overly complex and fits the training data too closely, leading to high variance. The optimal model structure lies in the sweet spot where the total error (bias + variance) is minimized.

One crucial aspect of the identification process in modelling, regardless of the specific technique used, is defining a reduced-order model following the Occam’s razor principle [172]. Model complexity refers to the capacity of a model to capture the underlying patterns in the data. A model with high complexity can fit almost any data perfectly, including the noise, which can lead to *overfitting*. On the contrary, a model with low complexity may be too simplistic to capture the essential features of the data, leading to underfitting. The challenge lies in finding the right (optimal) level of complexity that results in the best predictive performance. This trade-off between model simplicity and performance highlights the need for a good understanding of the bias-variance dilemma [172] in the modelling step of the software sensor design [16]. It refers to the trade-off encountered when making statistical predictions, such as fitting a function, between the accuracy of the prediction and its precision. This trade-off is essentially between bias (the inverse of accuracy) and variance (the inverse of precision). The prediction error, which measures the difference between actual and predicted values, consists of three components (Figure 10):

- Bias Error: This reflects the model’s ability to fit the training data. A model with high bias (indicative of low complexity and low accuracy) tends to miss relevant relationships between input features and the target output, leading to underfitting (Figure 10).
- Variance Error: This indicates the model’s sensitivity to small fluctuations in the training data. A model with high variance (indicative of high complexity and low precision) captures the noise in the training data, making it less stable and resulting in overfitting. Such models perform well on training data but inadequately on unseen data due to their high complexity (Figure 10).
- Noise: This represents the irreducible error inherent in any biological data, which cannot be eliminated during the design process.

#### 5.2.2. Model Interpretability

During the design process of software sensors, it is also important to carefully consider the interpretability of the selected data-based model. Here comes another trade-off between model performance and these two concepts, which largely depends on the objectives of the software sensor design. Essentially, this trade-off involves deciding whether it is more important to achieve the best prediction performance or to extend the understanding of the system, potentially at the expense of some accuracy [16].

**Figure 11:**
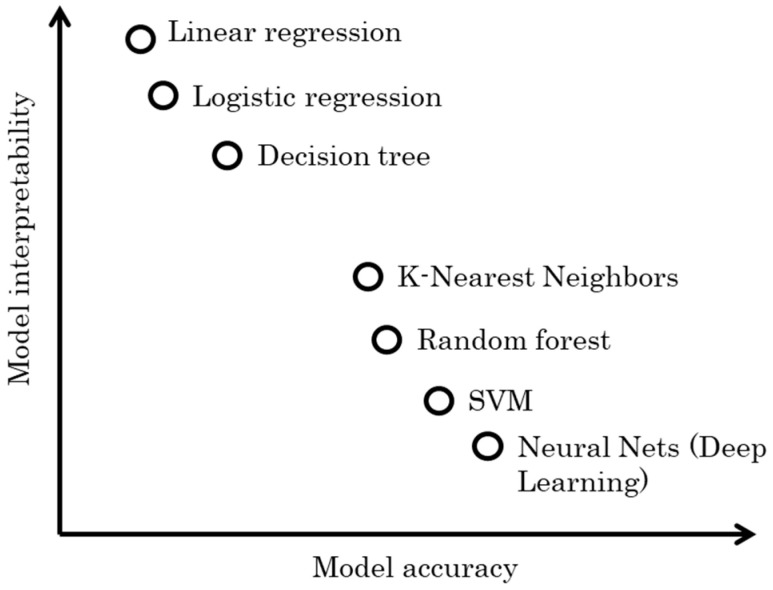
The interpretability–accuracy trade-off in in data-driven modelling: as model complexity increases, accuracy often improves but interpretability suffers.

Model interpretability refers to how each of the input (auxiliary) variables contributes to the prediction of the target output. An interpretable model provides clear insights into how input features are mathematically mapped to target outputs. Figure 11 provides a generalized view of the relationship between model interpretability and accuracy for some commonly used data-driven models. It is important to recognize that this comparison is a simplification, and actual performance can vary significantly depending on specific datasets and the problem domain. Take, for example, the following linear regression model [52], which is a common example of an interpretable model:

$$Y=a_{0}+a_{1}X_{1}+a_{2}X_{2}+\cdots +a_{n}X_{n}+e,$$

where Y, the target output (e.g., thermal comfort), is directly related to the input feature variables {$X_{1},X_{2},\ldots ,\;X_{n}$}, such as heart rate, skin temperature, and air temperature [52], and the coefficients {$a_{1},a_{2},\ldots ,\;a_{n}$} quantifies the contribution of each input variable. Each coefficient in the linear regression model directly indicates the impact of a corresponding input on the target output, making the model highly interpretable and easy to understand for stakeholders.

Generally, in scenarios where transparency and comprehensibility are crucial, such as in clinical diagnostics and exploratory research, highly interpretable models are preferred. However, in cases where maximizing prediction accuracy is crucial, more complex models like deep neural networks (DNNs) are often employed. DNNs involve data passing through multiple layers of multiplications with learned weights and non-linear transformations. A single prediction may result from millions of mathematical operations, rendering such models almost impossible to be fully interpreted.

## 6. Summary

This review has explored the potential of software sensors in revolutionizing the management and control of complex biosystems. Our primary objective was to demonstrate the advantages of this approach in overcoming the inherent challenges posed by the complexity, variability, and uncertainty of biological processes. We highlighted how software sensors, by combining data from traditional sensors with advanced computational models, can indirectly infer crucial variables that are difficult or impossible to measure directly.

Biosystems, which encompass a wide range of complexities from cellular processes to ecological interactions, are characterized by their time-varying, inherent nonlinearity and individual variability. Hence, the ultimate goal of biosystem monitoring is to uncover underlying mechanisms, identify potential issues, and optimize conditions for enhanced efficiency and productivity. This is particularly relevant in modern industrial applications such as agriculture and biotechnology, where real-time decision-making can significantly impact productivity and sustainability.

Despite the advancements in modern hardware sensors, the article highlights a critical challenge: what is typically measurable may not align with what needs to be monitored. Traditional sensing approaches often fall short in capturing all the necessary data due to difficulties related to physical accessibility or the high costs associated with advanced measuring techniques. Meanwhile, there is a specific need for a deeper understanding of nuanced behaviours and emergent properties of these systems, such as stress levels, health status, and overall wellbeing.

This article emphasizes that software sensors can effectively address these gaps by combining data from hardware sensors as auxiliary variables with estimations models and computational algorithms, enabling the accurate inference of target variables that are challenging to measure directly. The integration of enhanced sensing technologies and advanced computational methods has empowered significant advancements in the capabilities of software sensors, allowing for the continuous and unobtrusive monitoring of biosystems. The model-based monitoring and control framework allows for greater predictive power and responsiveness, essential for effective decision-making.

This article provides a brief overview of the common methodologies for designing software sensors with the main focus being on the estimation algorithm and modelling step. It provides an overview of various modelling approaches, distinguishing the main differences between hypothetico-deductive (mechanistic) models and inductive (data-driven) models such as linear regression, support vector machines, and neural networks.

Moreover, this article outlines the practical applications of software sensors in diverse fields, from environmental monitoring to precision agriculture and healthcare. The article presents three case studies, showcasing in detail three real-world applications of software sensors in biosystem monitoring and control. In the first case study, the focus is on non-invasive disease monitoring in animals, which demonstrates how software sensors can provide early warnings for respiratory infections by analyzing sound signals and cough patterns. Additionally, these sensors show remarkable versatility in multi-sensor fusion applications, allowing them to operate with various hardware sensors to enhance fault detection and minimize uncertainty. The second case study further illustrates this potential, illustrating their ability to integrate data from different hardware tools, such as satellite imagery and hyperspectral signals, to accurately predict target variables, namely, soil organic carbon (SOC) levels. Finally, the third case study presents the use of data assimilation techniques, such as Kalman filter, in automated greenhouse control. This example demonstrates how software sensors can optimize environmental conditions for crop growth, ultimately leading to improved yields and greater resource efficiency.

While the article thoroughly discusses the advantages of software sensors, it also addresses various considerations essential to their design process. Challenges related to the biological systems themselves, such as complexity, time-variability, and individual differences, are discussed, along with commonly used computational techniques to mitigate these issues. Additionally, this article outlines the challenges inherent in data-driven modelling approach, particularly the trade-off between model complexity and performance. The bias-variance dilemma is discussed as a key concern for software sensor designers.

Another considerable trade-off highlighted is between model performance and interpretability. Simpler models like linear regression offer high interpretability but may fail to capture complex patterns as effectively as more advanced models like deep neural networks, which, despite their higher accuracy, suffer from poor interpretability. Ultimately, selecting the most suitable modelling technique should align with the specific design objectives of the software sensor, balancing accuracy, interpretability, and the ability to adapt to the dynamic nature of biological systems.

In conclusion, software sensors represent a significant advancement in the monitoring and control of biosystems, enabling better decision-making and optimization through the integration of advanced hardware technologies and computational techniques. As we continue to develop these technologies, the collaboration between interdisciplinary teams, including data scientists, biologists, and engineers, will be crucial to addressing the complexities and challenges of modern biosystems.

## Data Availability

The original contributions presented in the study are included in the article, further inquiries can be directed to the corresponding author.
